# The influence of a patient counseling training session on pharmacy students’ self-perceived communication skills, confidence levels, and attitudes about communication skills training

**DOI:** 10.1186/s12909-019-1607-x

**Published:** 2019-05-28

**Authors:** Hye Kyung Jin, So Hyun Park, Ji Eun Kang, Kyung Suk Choi, Hong Ah. Kim, Min Seon Jeon, Sandy Jeong Rhie

**Affiliations:** 1 College of Pharmacy, Ewha Womans University, Seoul, 03760 Republic of Korea; 2 College of Pharmacy and Division of Life and Pharmaceutical Sciences Graduate School, Ewha Womans University, Seoul, 03760 Republic of Korea; 3 Department of Pharmacy, National Medical Center, Seoul, 04564 Republic of Korea; 4 Department of Pharmacy, Seoul National University Bundang Hospital, Seongnam, 13620 Republic of Korea

**Keywords:** Attitudes, Communication skills, Confidence, Pharmacy education, Pharmacy student

## Abstract

**Background:**

The ability to communicate effectively is an essential skill for a pharmacist. However, the curricula of most pharmacy schools in South Korea do not include communication skills training (CST). This study aims to evaluate the effects of CST in pharmacy education.

**Methods:**

This study was a comparison of pre- and post-intervention surveys completed by sixty fifth-year pharmacy students who participated in communication skills and patient counseling training during the spring 2017 semester. The students were asked to respond to 49 questions addressing 4 self-assessment categories: communication skills (24), attitudes (19), and confidence levels (2) at the beginning and end of the CST, and their perception of CST (4) after completing the course. The training session included lectures, small group work, role play, videos, and performance feedback by a tutor. Data were analyzed using the paired *t*-test with Bonferroni’s correction for multiple comparisons. The open-ended questions were analyzed using inductive content analysis.

**Results:**

The pharmacy students’ self-assessment of their communication skills, attitudes toward the communication course, and confidence levels showed significant improvement after the CST. Most students (96.7%) indicated the necessity of a pharmacy communication curriculum. They responded that CST is helpful for effective communication with patients (33.3%) and other healthcare professionals (31.7%). Role-playing was reported as the most preferred learning method (58.3%).

**Conclusions:**

CST significantly impacted pharmacy students’ skills, attitudes, and confidence levels related to communication skills and patient counseling. These findings indicate that communications training should be included in the regular curriculum of pharmacy schools.

## Background

Effective communication skills are vital in the pharmacy profession. Appropriate and effective communications with patients and other healthcare providers (e.g., doctors, nurses, and pharmacists) about drug therapy and patient care are associated with fewer medication errors, improvement of patient’s understanding of treatment, medication adherence, and optimal health outcomes [1–4]. Moreover, as pharmaceutical care has become more patient-centered, the communication abilities of pharmacists have become more important. The Center for the Advancement of Pharmaceutical Education (CAPE) and the Accreditation Council for Pharmacy Education (ACPE) have emphasized communication skills training (CST) to develop skills that are necessary in the health care environment [5, 6]. Pharmacists require effective communication skills to provide patient-centered care within an interprofessional health care team [7, 8]. In other words, developing competence in communication is as important as acquiring pharmaceutical knowledge and clinical skills.

Pharmacy educators have used a variety of methods to teach communication skills, including didactic lectures, small group work, role-playing, simulated patient interactions, and video reviews [9, 10]. In addition, motivational interviewing that focuses on patient-centered counseling has recently received increased attention in pharmacy education [5, 11]. However, despite the widespread acceptance of the growing importance of CST in the pharmacy curriculum, challenges remain in providing students with the essential knowledge and skills to become competent communicators because the students themselves may not recognize their communication deficiencies. Students tend to have flawed self-assessment skills, such as overestimating or underestimating their ability to communicate [12, 13]. This tendency may result from lack of knowledge and limited opportunities to receive constructive and objective feedback on their communication performance. However, several studies have shown that effective communication and counseling skills can be taught and practiced [9, 14–17]. Additionally, early and repeated learning of these skills is beneficial to students, as it allows time to continue developing and refining the skills throughout their pharmacy training [17]. For these reasons, some pharmacy educators agree that the first year is the optimal time to introduce communication skills [18].

Despite the increasing acknowledgment of the importance of communication skills and related educational programs, reflected in the global trends in pharmacy education, relatively few students in South Korea have the opportunity to attend a communications course during their pharmacy training. Also, no studies evaluating the effectiveness or impact of such training has been conducted. The Ewha Womans University College of Pharmacy curriculum attempts to foster the development of effective communication skills focusing on patient-centered care through an experimental laboratory course. Therefore, the aim of this study was to provide students the opportunity to participate in well-designed communication training, and to explore their attitudes about the value of a communications course in the pharmacy curriculum, their perceived differences in the patient communication skills and their level of confidence in the ability to communicate with patients.

## Methods

### Study design and participants

This study was based on a pre-post interventional design. Half of the fifth-year undergraduate pharmacy students (*n* = 64) at the Ewha Womans University College of Pharmacy were required to participate in a course entitled Pharmaceutical Experiment Laboratory VI during the spring 2017 semester. None of the students had previous training or had completed a course in communication skills before the study. The students were asked to complete a survey before the CST and another survey after completing the training containing open-ended questions with a free text response format that solicited students’ suggestions for future courses. The participants’ responses were anonymized. Informed consent was obtained from each participant. The study was approved by the Institutional Review Board of Ewha Womans University (Number: 138–7).

### Description of the communication skills training

**Fig. 1:**
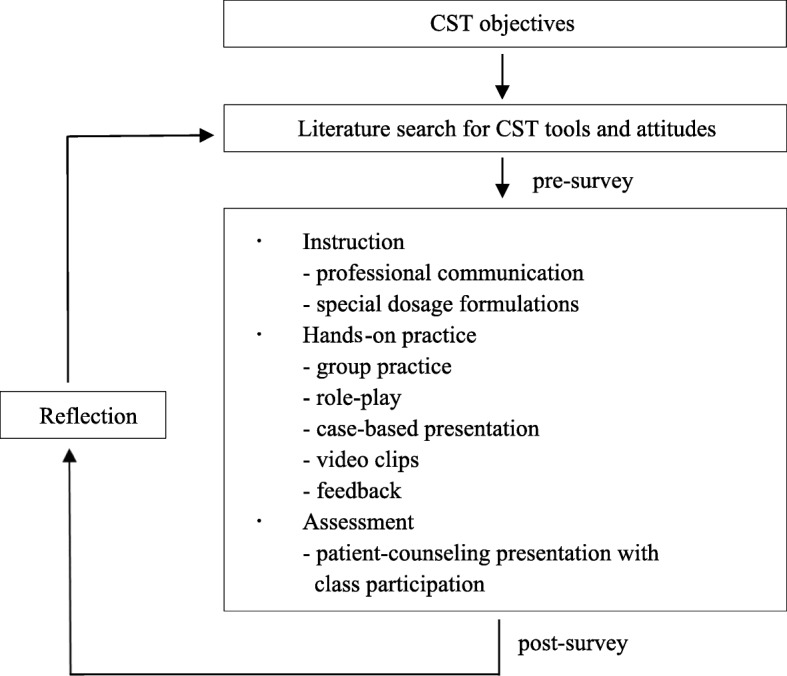
CST context and process. Note: *CST* Communication skills training

The process used to conduct the CST is summarized in Fig. 1. The objective of the CST was based on Bloom’s taxonomy [19], which addresses the three domains of learning: cognitive, affective, and psychomotor. We focused on measuring aspects of the affective domain (e.g., communication skills and attitudes). The training session focused on hands-on patient counseling and communication training for 3 h in the 10th-week of the semester-long course. The training session was divided into instruction, practice, and assessments addressing the themes of communication skills with a focus on patient counseling.

#### Part A: instruction in professional communication skills

All students attended a 50-min didactic session. The lecture addressed the importance of communication skills in the pharmacy profession, the benefits of effective communication, the types and classification of communication, general patient interview and counseling techniques, verbal and nonverbal communication skills, empathy, active listening, and interprofessional communication.

#### Parts B & C: patient assessments and counseling about special dosage forms

Sixty-four students were organized into small groups of 3 classes; each class participated in the training session on a different day. Each class was further divided into 5 groups of 4 or 5 students. An instructor facilitated the CST using lectures, video clips, group practice, role-playing, and case-based presentations. Each group was given time to prepare role plays for a counseling presentation on special dosage forms (e.g., rectal suppositories, otic solutions, ophthalmic solutions/ointments, nasal solution/sprays, various inhalers, and injectable pens) acting as a patient or pharmacist. Afterward, 2 students per group were randomly selected to perform the roles for 5 min using the format of the American Pharmacists Association (APhA) National Patient Counseling (NPC) Competition. This demonstration was followed by immediate feedback from the instructor and classmates regarding the students’ communication skills. The demonstrations were video-recorded and uploaded to a course website to enable students to review their performance any time.

### Instruments

The survey consisted of questions about the students’ demographic characteristics; their communication skills, attitudes, and confidence; the usefulness of the teaching methods; and their suggestions for future courses. The reliability of each part of the survey tool was assessed with Cronbach’s alpha.

The survey questions on communication skills were based on the Calgary-Cambridge Observation Guide modified by the Centre for Pharmacy Postgraduate Education (CPPE) of the National Health System (NHS) [20–22]. After consulting with two pharmacy experts to enhance its clarity, we removed the items unrelated to pharmacy such as contacting the patient about physician follow-up. The final version of the questionnaire had 24 questions, including the six domains of initiating the session, gathering information, providing structure, building a relationship, explanation and planning, and closing the session, all of which were rated on a 7-point Likert scale (1 = very strongly disagree to 7 = very strongly agree).

The pharmacy students’ attitudes toward learning communication skills were measured with a modified version of the Communication Skills Attitudes Scale (CSAS) [23]. We removed seven items and modified some terminology to match our study focus. For example, “Communication skills teaching would have a better image if it sounded more like a science subject” was deemed confusing to the students and was removed. Additionally, “Learning communication skills is fun” was excluded due to conceptual overlap with the item “Learning communication skills is interesting” when translated in Korean. Further, “physician” was changed to “pharmacist,” and the phrase “is applicable to learning medicine” was modified to “is applicable to learning pharmacy.” The final version of the modified CSAS questionnaire consisted of 2 subscales of 19 items scored on a 5-point Likert scale (1 = strongly disagree to 5 = strongly agree; questions shown in Table 3). Ten items contributed to the positive attitude subscale (PAS) score (e.g., “In order to be a good pharmacist, I must have good communication skills”), and the remaining nine items contributed to the negative attitude subscale (NAS) score (e.g., “I don’t need good communication skills to be a pharmacist”). All negative CSAS items were reverse-coded for analysis so that higher scores indicated a more positive attitude or willingness to learn communication skills. The Calgary-Cambridge guide and the modified CSAS were translated from the original English versions into Korean by independent researchers and then back-translated into English until a consensus was reached by further discussion. If a consensus could not be reached, alternative translations of items were discussed among the research team. The comprehensibility and the readability of these items were assessed. Cronbach’s alphas were 0.932 and 0.844 for the communication skills and attitudes scales, respectively.

The degrees of confidence concerning “communicating with patients” and “counseling on special dosage forms” were assessed with a pre- and post-survey scored on a 5-point Likert scale (1 = not confident to 5 = totally confident). The necessity of the CST was assessed with two questions in the post-survey using a 5-point Likert scale (1 = strongly disagree to 5 = strongly agree). Students were also asked to respond to the following open-ended questions: (1) Why is learning communication skills necessary? and (2) What specific contents would you add to the CST?

### Data analysis

The quantitative data were analyzed with descriptive statistics. To analyze the differences between the mean scores of the students’ self-assessed awareness, attitude, communication skills, and confidence level scores, we performed a paired *t*-test with SPSS version 24.0 (IBM Co., Armonk, NY, USA). All the analyses were two-sided, and *p* < 0.05 was considered statistically significant. In the case of multiple comparisons, Bonferroni’s corrections were applied, and our more conservative criterion for significance was *p* < 0.001. Inductive content analysis was used to analyze answers to open-ended questions [24]. One investigator coded and categorized the responses to the open-ended questions. Two investigators reviewed the coding and categories. When differences in coding occurred, they were discussed until a consensus was reached, and the frequencies of the coded categories were then recorded.

## Results

### Study participants

Sixty of the 64 students (93.8%) consented to participate and completed the surveys. Most of the participants (96.7%) responded that a communications course was necessary and all participants (100%) stated that such training was required for their professional careers. The top three reasons for learning communication skills were, “It would help me communicate effectively with patients” (33.3%), “It would support effective communication with other healthcare professionals or colleagues” (31.7%), and “Communication is a part of a pharmacist’s job” (21.7%) (Table 1).

**Table 1:** Demographic and educational characteristics of the fifth-year pharmacy students participating in communication skills training (*n* = 60)

Characteristic	n (%)
Gender	
Female	60 (100)
Age, years (range 22–32), mean ± SD	24.18 ± 1.90
22–24	42 (70.0)
25–27	15 (25.0)
28–30	2 (3.3)
31 or older	1 (1.7)
Students’ self-rating of communication skills	
Very poor	6 (10.0)
Poor	18 (30.0)
Fair	28 (46.7)
Good	8 (13.3)
Very good	0 (0.0)
Students’ assessment that their communication skills require improvement^a^	
Agree	11 (18.3)
Strongly agree	49 (81.7)
Communication skills course is needed in a pharmacy curriculum^b^	
Neutral	2 (3.3)
Agree	22 (36.7)
Strongly agree	36 (60.0)
Communication skills course is needed for pharmacy students to improve themselves professionally^c^	
Agree	24 (40.0)
Strongly agree	36 (60.0)
Reasons for learning communication skills^d^	
It helps to communicate with patients	20 (33.3)
It helps to effectively communicate with physicians, nurses, or colleagues	19 (31.7)
Communication is a part of a pharmacist’s job	13 (21.7)
It helps to improve medication adherence	10 (16.7)
It helps to deliver accurate information to patients	5 (8.3)
It helps to build trust between patients and pharmacists	4 (6.7)
It helps to better understand patients’ problems	3 (5.0)

SD standard deviation

^a^Other response options were “strongly disagree” (*n* = 0), “disagree” (*n* = 0), and “neutral” (*n* = 0)

^b^Other response options were “strongly disagree” (*n* = 0) and “disagree” (*n* = 0)

^c^Other response options were “strongly disagree” (*n* = 0), “disagree” (*n* = 0), and “neutral” (*n* = 0)

^d^Percentage does not equal to 100% because the respondents were allowed to choose more than one option

### Self-assessed communication skills, attitudes, and confidence

Total mean scores with standard deviation were as follows for their communication skills, attitudes toward learning communication skills, confidence in their ability to communicate with patients, and confidence in their ability to perform patient counseling for special dosage formulations, respectively: pre, 4.64 (0.78) vs. post, 5.47 (0.72) *p* < 0.001 (Table 2); pre, 4.00 (0.45) vs. post, 4.22 (0.45) *p* < 0.001 (Table 3); pre, 2.57 (0.98) vs. post, 4.18 (0.89); *p* < 0.001 (Table 4); and pre, 1.85 (0.80) vs. post, 4.30 (0.70); *p* < 0.001 (Table 4).

**Table 2:** Comparison of the students’ self-assessed communication skills by category

Category and contents^a^	Pre (*n* = 60) mean (SD)	Post (*n* = 60) mean (SD)	t	*p*-value
Initiating the session				
Prepare yourself for the consultation	4.45 (1.48)	5.60 (0.99)	−5.556	< 0.001
Introduce yourself and welcome the patient	4.92 (1.45)	5.77 (1.03)	−4.375	< 0.001
Establish an initial rapport with the patient	4.68 (1.47)	5.48 (1.08)	−3.892	< 0.001
Identify the reason for the consultation	4.73 (1.33)	5.60 (1.01)	−4.541	< 0.001
Gathering information				
Explore the patient’s issues through effective questioning and listening skills	4.82 (1.13)	5.53 (0.91)	−4.441	< 0.001
Obtain the patient’s perspective	4.77 (1.11)	5.42 (1.01)	−3.660	0.001
Use concise, easily understood questions and comments and avoid or adequately explain jargon	4.55 (1.24)	5.53 (1.16)	−5.073	< 0.001
Explanation and planning				
Provide the correct type of information in an appropriate manner suitable for the patient	4.32 (1.02)	5.48 (0.89)	−7.000	< 0.001
Aid accurate recall and understanding	4.52 (1.08)	5.58 (0.87)	−6.231	< 0.001
Achieve a shared understanding, incorporating the patient’s perspective	4.55 (0.98)	5.38 (0.88)	−5.275	< 0.001
Develop an action plan that involves shared decision-making	4.30 (1.23)	5.15 (0.97)	−4.703	< 0.001
Providing structure to the consultation				
Agree with the agenda	4.67 (1.20)	5.63 (0.88)	−5.296	< 0.001
Summarize and recall throughout the consultation to check understanding	4.75 (1.37)	5.77 (0.85)	−5.131	< 0.001
Use signposts and transitional statements to progress from one part of the consultation to the next	4.23 (1.23)	5.22 (1.06)	−5.765	< 0.001
Apply a logical structure	4.20 (1.25)	5.25 (1.14)	−5.730	< 0.001
Adhere to the time limit	4.65 (1.23)	5.18 (1.00)	−2.862	0.006
Building a relationship				
Continue to build rapport throughout the consultation	4.90 (1.31)	5.53 (0.98)	−3.332	0.001
Show empathy	5.15 (1.34)	5.58 (1.12)	−2.327	0.023
Share in the discussion as a partnership	4.63 (1.30)	5.32 (1.05)	−3.637	0.001
Use open body language and appropriate eye contact	4.53 (1.35)	5.47 (1.03)	−5.736	< 0.001
Closing the session				
Summarize the key ideas	4.83 (1.03)	5.33 (1.07)	−2.774	0.007
Make a final contract with the patient to agree with the action plan	4.65 (0.95)	5.37 (0.94)	−4.541	< 0.001
Establish contingency plans in case the plan does not proceed as designed (safety-netting)	4.20 (1.30)	5.22 (1.04)	−4.859	< 0.001
Asks if the patient has any questions or other items they would like to discuss	5.40 (1.30)	5.98 (0.93)	−3.014	0.004

*SD* standard deviation

^a^7–point Likert scale for all items, pre and post (1 = very strongly disagree; 7 = very strongly agree)

**Table 3:** Comparison of the students’ self-assessed attitudes towards learning communication skills

Items^a,b^	Pre (*n* = 60) mean (SD)	Post (*n* = 60) mean (SD)	t	*p-*value
(1) In order to be a good pharmacist, I must have good communication skills	4.40 (0.64)	4.78 (0.45)	−4.638	< 0.001
(2) I can’t see the point of learning communication skills(R)	4.65 (0.61)	4.68 (0.72)	−0.340	0.735
(3) Developing my communication skills is just as important as developing my knowledge of pharmacy	3.90 (0.90)	4.43 (0.70)	−4.000	< 0.001
(4) Learning communication skills has helped or will help me respect patients	4.37 (0.69)	4.58 (0.62)	−2.205	0.031
(5) I haven’t time to learn communication skills(R)	2.62 (1.28)	3.22 (1.21)	−2.992	0.004
(6) Learning communication skills is interesting	4.02 (0.83)	4.15 (0.78)	−1.158	0.252
(7) I can’t be bothered to turn up to sessions on communication skills(R)	4.17 (1.09)	4.35 (0.86)	−1.260	0.213
(8) Learning communication skills has improved my ability to communicate with patients	4.17 (0.75)	4.53 (0.60)	−3.493	0.001
(9) Communication skills teaching states the obvious and then complicates it(R)	3.12 (1.20)	3.67 (1.07)	−3.325	0.002
(10) Learning communication skills is too easy(R)	3.80 (0.90)	3.63 (0.90)	1.371	0.176
(11) Learning communication skills has helped or will help me respect my colleagues	4.09 (0.82)	4.21 (0.67)	−1.069	0.290
(12) I find it difficult to trust information about communication skills provided by non-clinical lecturers(R)	3.87 (1.08)	3.73 (1.10)	0.841	0.404
(13) Learning communication skills has helped or will help me recognize patients’ rights regarding confidentiality and informed consent	3.78 (0.94)	4.23 (0.65)	−3.227	0.002
(14) I don’t need good communication skills to be a pharmacist(R)	4.60 (0.49)	4.63 (0.69)	−0.362	0.718
(15) I find it hard to admit having some problems with my communication skills(R)	3.67 (1.00)	3.73 (0.86)	−0.489	0.626
(16) I think it’s really useful to learn communication skills during pharmacy training	4.18 (0.87)	4.38 (0.67)	−1.802	0.077
(17) Learning communication skills is applicable to learning pharmacy	3.97 (0.86)	4.38 (0.76)	−3.799	< 0.001
(18) Learning communication skills is important because my ability to communicate is a lifelong skill	4.30 (0.77)	4.43 (0.79)	−1.211	0.231
(19) Communication skills education should be left to psychology students, not pharmacy students(R)	4.43 (0.87)	4.42 (0.81)	0.131	0.896

*SD* standard deviation

^a^Items marked (R) are negative, and the score was reversed before the analysis. Therefore, higher scores indicate more positive attitudes about learning communication skills

^b^Scores based on a 5-point Likert scale for all items; pre and post (1 = strongly disagree; 5 = strongly agree)

**Table 4:** Comparison of the students’ confidence in their communication skills

How confident are you in your ability regarding:^a^	Pre (*n* = 60) mean (SD)	Post (*n* = 60) mean (SD)	t	*p-*value
Communicating with patients	2.57 (0.98)	4.18 (0.89)	−10.018	< 0.001
Patient counseling on special dosage forms	1.85 (0.80)	4.30 (0.70)	−17.568	< 0.001

*SD* standard deviation

^a^Scores based on a 5-point Likert scale for all items, pre and post (1 = not at all confident; 5 = extremely confident)

### Students’ perceptions of learning methods

Role play (58.3%) was rated as the most preferred learning method, followed by feedback (18.3%), videotaping and review (11.7%), lectures (8.3%), and group activities (3.3%).

### Students’ suggestions for future courses

Students wanted to learn communication skills for dealing with special populations or situations. The top four contents were, “Communicating with difficult patients” (31.7%), “Anxious or hostile patients” (26.7%), “Building trust” (20.0%), and “Delivering bad news to patients and families” (18.3%).

Moreover, most students (74.0%) indicated that attending the communications course early in their training would prevent the acquisition of negative communication habits and skills.

## Discussion

Pharmacists’ roles and responsibilities have become more patient-centered, and effective communication skills are required to satisfy the health needs of diverse patient populations. The pharmacy education system in Korea has changed from a 4-year BS degree program to a 6-year Doctor of Pharmacy (PharmD) degree program in response to the need for patient-centered professionalism. However, the lack of communication skills training persists. The results of this study show that learning communication skills for patient counseling in peer role-playing sessions can improve pharmacy students’ self-reported communication skills, attitudes about learning these skills, and confidence levels.

To our knowledge, this is the first study to examine pharmacy students’ perceptions of the impact of learning communication skills in South Korea. There is a paucity of communication skills courses in the pharmacy curricula in South Korea, possibly due to a lack of understanding of the value of communication in pharmacy practice. Faculty members tend to believe that communication is relevant only to patient counseling [10]. In addition, most pharmacy schools emphasize science-based curricula. The results of this study suggest that pharmacy students would benefit from ample opportunities to learn and practice communication skills within suitable training programs. Recently, team-based medical care has been increasing internationally, and the participation of pharmacists has been expanding [25–29]. This suggests that the collaboration and communication among various healthcare professionals in the hospital is also very important along with effective communication with patients. Effective interprofessional communication in healthcare enhances cohesion among members, the cooperative spirit of each member, and team efficiency, thereby reducing conflicts between regions [3, 30]. Moreover, it plays an important role in problem solving and reasonable decision making, which helps to improve the job satisfaction of pharmacists by ensuring they make the right decisions and efficiently circulate the information required for patient-centered care [31, 32]. Hence, pharmacy educators and curriculum planners in South Korea should consider including communication courses in the undergraduate pharmacy training curriculum to prepare students for their professional roles in more patient-centered care environments.

Although this study was limited in scope due to time restrictions, the significant self-perceived improvements in communication skills as a result of the single training session were encouraging. However, Aspegren asserts that communication skills can be taught and are learned, but training for more than one day is more effective, and such training should be repeated to maintain these skills [33]. Therefore, it would be beneficial to integrate communication skills within the basic structure of the pharmacy curriculum rather than offering it as a single course. Adrian and colleagues recently demonstrated improvements in students’ oral and written communication skills after participating in a course that included case scenarios role-play [34]. Rogers and King also found similar results in a first-year PharmD student course that included role-playing exercises [18]. Interestingly, after the session, students scored significantly higher on several items addressing the emotional aspects of communication skills, such as the “show empathy” item. This result was similar to those of previous reports demonstrating that empathy can be taught [35, 36].

Although the total mean score of the self-assessed attitude items improved significantly, only 3 of the survey questions about self-assessed attitudes showed significant changes, which was different from the results of the self-assessed communication skills and confidence level questions. One possible explanation relates to the short duration of training, which may not have been sufficiently impactful to change deep-rooted attitudes or behaviors related to communication. This result indicates that considerable time and effort may be necessary to initiate a change in students’ existing attitudes. The response to the statement “I find it difficult to trust information about communication skills given to me by non-clinical lecturers” was negative. This result suggests that pharmacy students think that communication skills are important but that a nonclinical lecturer is inappropriate for teaching these skills, resulting in lower motivations among students. A relatively recent study found that most pharmacy students belong to the current generation that prefers active and experiential learning, online and virtual resources, over traditional lectures [37, 38]. Pharmacy schools in Australia have incorporated communication skills into their undergraduate curricula [39] and have developed technological resources for teaching communication [38]. Therefore, a communication method that stimulates interest may promote active participation in class and provide new insights for communication.

Students’ confidence in their ability to provide patient counseling on special dosage forms increased significantly after completing the CST, which accords with the results of previous studies [40, 41]. A national survey conducted in US pharmacy schools showed that early intervention was required to overcome communication issues [10] and similar findings were reported in studies of UK medical and dental students [23, 42]. Adrian et al. reported that shifting the communications skills course from the second year to the first year enabled a better understanding of communication skills and provided more time to practice skills [34]. Based on these results, we believe that exposing students to this training at an earlier stage in their education would help build their confidence to a greater extent and would produce competent future pharmacists capable of providing appropriate patient counseling.

This study has some limitations. First, the questionnaire used self-assessment methods rather than objectively assessed skills. Development of objective and validated tools is warranted to confirm whether the improvement in communication skills is maintained and whether these skills are used in practice with actual patients. Also, all the study participants were women as the study was conducted at a women’s university, and the study only included students from one institution; these factors limit the generalizability of the results.

## Conclusions

Pharmacy students perceived that the communication training session was useful not only for improving their communication skills but also for increasing their confidence in their communication abilities and improving their attitude towards learning communication skills. We believe that these findings provide insight into an area about which little is currently known in the context of South Korean pharmacy education.

## Abbreviations

ACPE: Accreditation Council for Pharmacy Education
APhA: American Pharmacists Association
CAPE: Center for the Advancement of Pharmaceutical Education
CPPE: Centre for Pharmacy Postgraduate Education
CSAS: Communication Skills Attitudes Scale
CST: Communication Skills Training
IPPE: Introductory Pharmacy Practice Experience
NAS: Negative Attitude Subscale
NHS: National Health System
PAS: Positive Attitude Subscale
SP: Standardized or Simulated Patient
